# Three-dimensional-printed patient-specific instrumentation is an accurate tool to reproduce femoral bone tunnels in multiple-ligament knee injuries

**DOI:** 10.1007/s00264-023-05712-1

**Published:** 2023-02-17

**Authors:** Núria Fernández-Poch, Ferran Fillat-Gomà, Laia Martínez-Carreres, Sergi Coderch-Navarro, Christian Yela-Verdú, Sonia Carbó-Cedán, Xavier Pelfort

**Affiliations:** 1 Orthopaedics Department, Parc Taulí Hospital Universitari, Institut d’Investigació I Innovació Parc Taulí I3PT, Universitat Autònoma de Barcelona, Department of Surgery, Sabadell, Spain; 2 3D Surgical Planning Lab., Parc Taulí Hospital Universitari, Institut d’Investigació I Innovació Parc Taulí I3PT, Universitat Autònoma de Barcelona, 08208 Sabadell, Spain; 3 Radiology Department, Parc Taulí Hospital Universitari, Institut d’Investigació I Innovació Parc Taulí I3PT, Universitat Autònoma de Barcelona, Sabadell, Spain

**Keywords:** Multiple-ligament reconstruction, Knee, Tunnels, 3D printing, Patient-specific instrumentation

## Abstract

**Purpose:**

Multiple-ligament knee reconstruction techniques often involve the creation of several bone tunnels for various reconstruction grafts. A critical step in this procedure is to avoid short tunnels or convergences among them. Currently, no specific template guide to reproduce these angulations has been reported in the literature, and the success of the technique still depends on the experience of the surgeon. The aim of this study is to analyze the accuracy and reliability of 3D-printed patient-specific instrumentation (PSI) for lateral and medial anatomical knee reconstructions.

**Methods:**

Ten cadaveric knees were scanned by computed tomography (CT). Using specific computer software, anatomical femoral attachments were identified: (1) on the lateral side the lateral collateral ligament (LCL) and the popliteal tendon (PT) and (2) on the medial side the medial collateral ligament (MCL) and the posterior oblique ligament (POL). Four bone tunnels were planned for each knee, and PSI with different directions were designed as templates to reproduce the planned tunnels during surgery. Twenty 3D-printed PSI were used: ten were tailored to the medial side for reconstructing MCL and POL tunnels, and the other ten were tailored to the lateral side for reconstructing LCL and PT tunnels. Postoperative CT scans were made for each cadaveric knee. The accuracy of the use of 3D-printed PSI was assessed by superimposing post-operative CT images onto pre-operative images and analyzing the deviation of tunnels performed based on the planning, specifically the entry point and the angular deviations.

**Results:**

The median entry point deviations for the tunnels were as follows: LCL tunnel, 1.88 mm (interquartile range (IQR) 2.2 mm); PT tunnel, 2.93 mm (*IQR* 1.17 mm); MCL tunnel, 1.93 mm (*IQR* 4.26 mm); and POL tunnel, 2.16 mm (*IQR* 2.39). The median angular deviations for the tunnels were as follows: LCL tunnel, 2.42° (*IQR* 6.49°); PT tunnel, 4.15° (*IQR* 6.68); MCL tunnel, 4.50° (*IQR* 6.34°); and POL tunnel, 4.69° (*IQR* 3.1°). No statistically significant differences were found in either the entry point or the angular deviation among the different bone tunnels.

**Conclusion:**

The use of 3D-printed PSI for lateral and medial anatomical knee reconstructions provides accurate and reproducible results and may be a promising tool for use in clinical practice.

## Introduction

Knee dislocation is a rare injury but has potentially devastating consequences for the injured patients [1, 2]. There is consensus in the literature that the surgical treatment of these lesions improves clinical outcomes compared to nonsurgical management [3–5]. In recent years, there has been a progressive evolution in surgical techniques toward a more anatomical reconstruction of the injured ligaments [6–9]. Most of these studies have reported better clinical and functional results if all ligament reconstructions were performed in a one-step surgery [10–12]. The reconstruction of these ligaments often involves the creation of several tunnels in a small area of the distal femur. Due to the limited bone mass, a critical step in these procedures is to avoid short tunnels or convergences among them because this can compromise the integrity of the graft [13, 14] and may result in damage to fixation devices, poor graft fixation, or intra-operative and post-operative femoral fractures [15–18]. Some authors have proposed performing these anatomical reconstructions following a specific recommendation on the direction of the bone tunnels to avoid all of these potential complications [19–21]. However, in clinical practice, it is difficult to perform them in an accurate and replicable manner because a specific template guide does not exist to reproduce these angulations. Then, the success of the surgery still depends on the experience of the surgeon. In recent years, indications for the use of 3D-printed patient-specific instrumentation (PSI) technology in orthopaedic surgery procedures have significantly increased, and high degrees of precision, accuracy, and reproducibility have been achieved [22, 23]. The purpose of the study was to analyze whether PSI technology may be an accurate tool to reproduce the entry point and direction of femoral bone tunnels for medial and lateral anatomical knee reconstructions based on pre-operative planning using a knee CT scan.

## Methods

This experimental surgery study, based on a human cadaveric model, received institutional review board approval registered CEIC number 2021/5027.

### Surgical planning and guide design

Pre-operative computed tomography (CT) scan of each cadaveric knee was performed using a Discovery PET/CT 690 system (GE Healthcare, USA) with the following characteristics: a minimum slice thickness of 0.625 mm (1 mm max.), contiguous or overlapping slices (no gaps allowed), a matrix size of 512 × 512, a voxel size of 0.6, and an anatomical region default kernel (standard or high resolution) of 90–120 kVp. The images were post-processed to a mesh-volume file, and specific segmentation of the region of interest was performed with Materialise Mimics 21.0 (Mimics Innovation Suite, Materialise MV, Belgium). Mesh-volume files were transferred to the design software 3-matic 13.0 (Mimics Innovation Suite, Materialise MV, Belgium) to conduct surgical planning and surgical guide design. For this purpose, the anatomical femoral attachments of the lateral collateral ligament (LCL) and popliteal tendon (PT) on the lateral side [24] and the medial collateral ligament (MCL) and posterior oblique ligament (POL) on the medial side [25] were first identified. Then, four bone tunnels were planned for each knee starting from the anatomical attachments of the LCL, PT, MCL, and POL applying different directions. Two personalized surgical guides were designed each knee to reproduce the planned tunnels during surgery: the first one for the LCL and PT and the second one for the MCL and POL. The direction of the tunnels was variable. The design criteria to be followed in all cases were as follows: (1) no coalescence of the planned tunnels, (2) no intra-articular invasion at the femorotibial level, and (3) no invasion of the femoral trochlea. This allowed us to analyze the degree of precision of the technique in different surgical scenarios.

### 3D printing of surgical guides

**Fig. 1:**
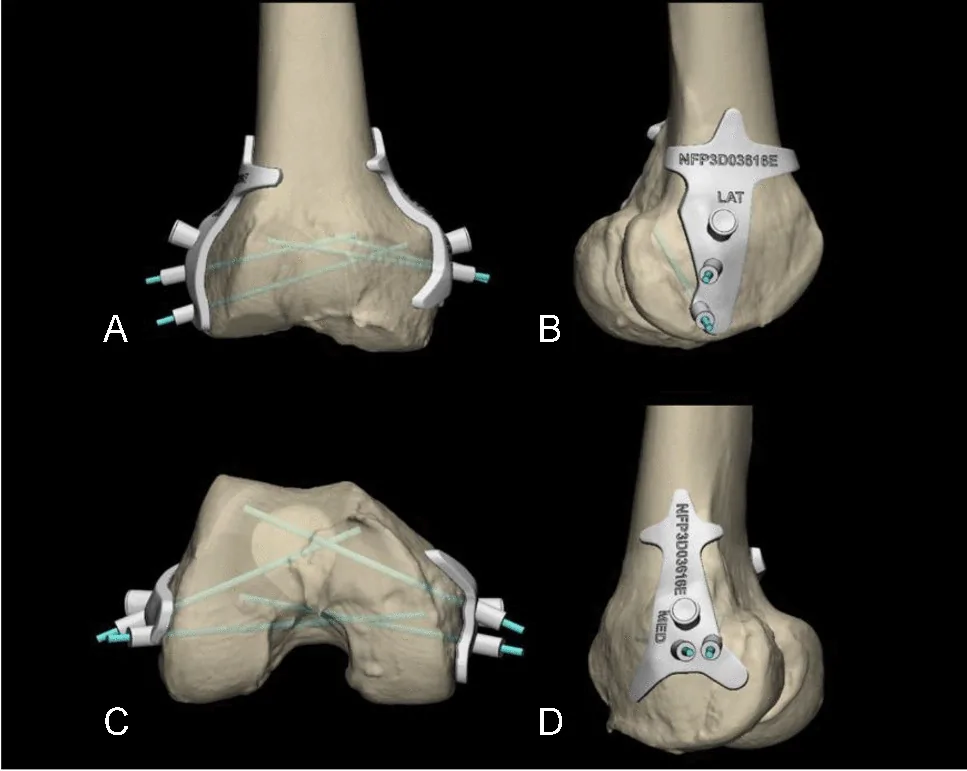
**A** Coronal, **B** lateral, **C** axial, and **D** medial view of surgical planning including PSI

Initially, the first cadaveric knee was used as a model to create different PSI guides until the optimal design was obtained for the correct application in the bone. Subsequently, eighteen 3D-printed PSI (9 for the medial side and 9 for the lateral side) specifically designed for each cadaveric knee (Fig. 1A–D) were printed internally in our center using polylactic acid (PLA) and polyvinyl alcohol (PVA) support with an Ultimaker 3/S5 printer (Ultimaker, Netherlands) by fused deposition modeling (FDM) technology and Ultimaker Cura 4.0 printing software (Ultimaker, Netherlands). During the design and creation of the guide, emphasis was placed on creating small-sized PSI that would fit well to avoid being too aggressive with soft tissue during the surgery. In the design process, a tolerance of 0.4 mm was applied. In this way, the guide fits perfectly into the bone edges, considering any remaining soft tissue.

### Surgical management

#### Lateral approach

A 5-cm lateral incision was performed. Then, the iliotibial band (ITB) was opened, and the approach was distally extended between the Gerdy tubercle and the fibular head. Dissection was performed in this location in the proximal and distal directions, exposing the lateral epicondyle until the FCL and PT femoral attachments were visualized. Then, minimal subperiosteal proximal dissection was performed to allow a correct adaptation of PSI to the femoral bone surface. To achieve this without damaging the remains of the ligament and capsule attachments, the guides were designed and printed allowing 0.4 mm of tolerance. Then, Kirschner wires were introduced across the whole guide. Finally, both tunnels were drilled with lengths of 25 mm and 8 mm in diameter after removing the PSI following the technique described by Laprade et al. [26]. The PSI design had a low profile, so if the position of Kirschner wires was too divergent, it was possible to break the guide in order to keep the Kirschner wires in place.

#### Medial approach

**Fig. 2:**
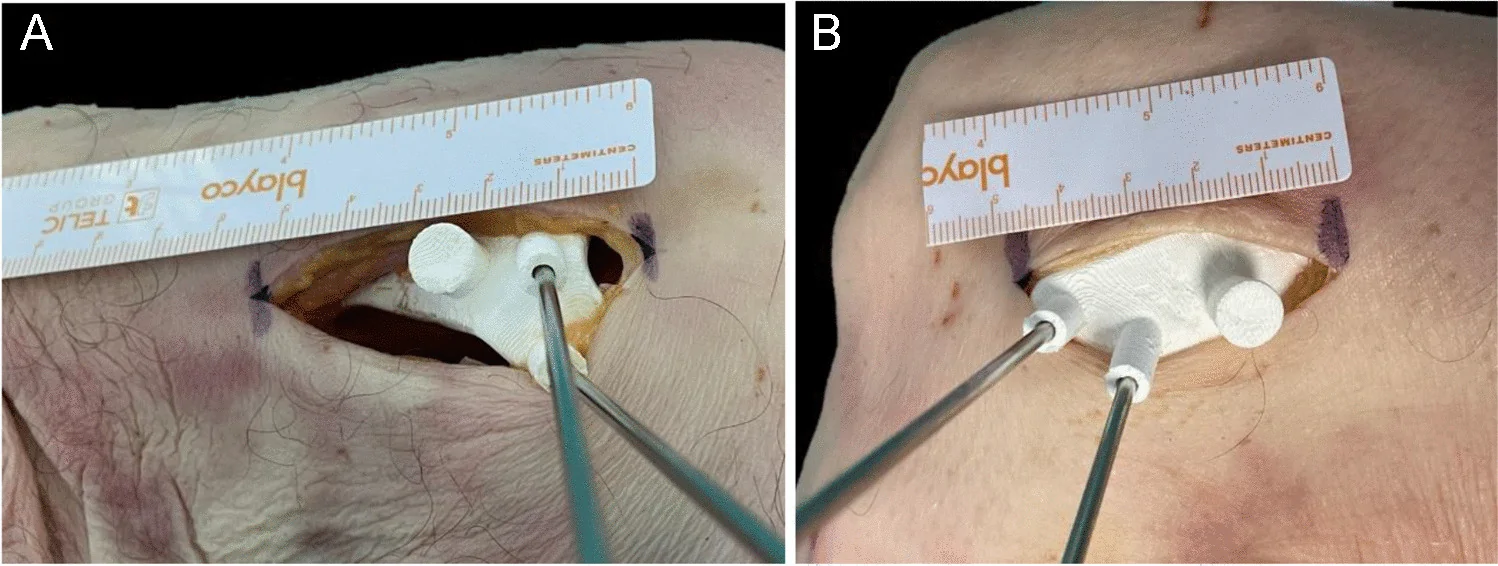
Medial (**A**) and lateral (**B**) approach. Correct PSI template positioning with Kirschner wires

**Fig. 3:**
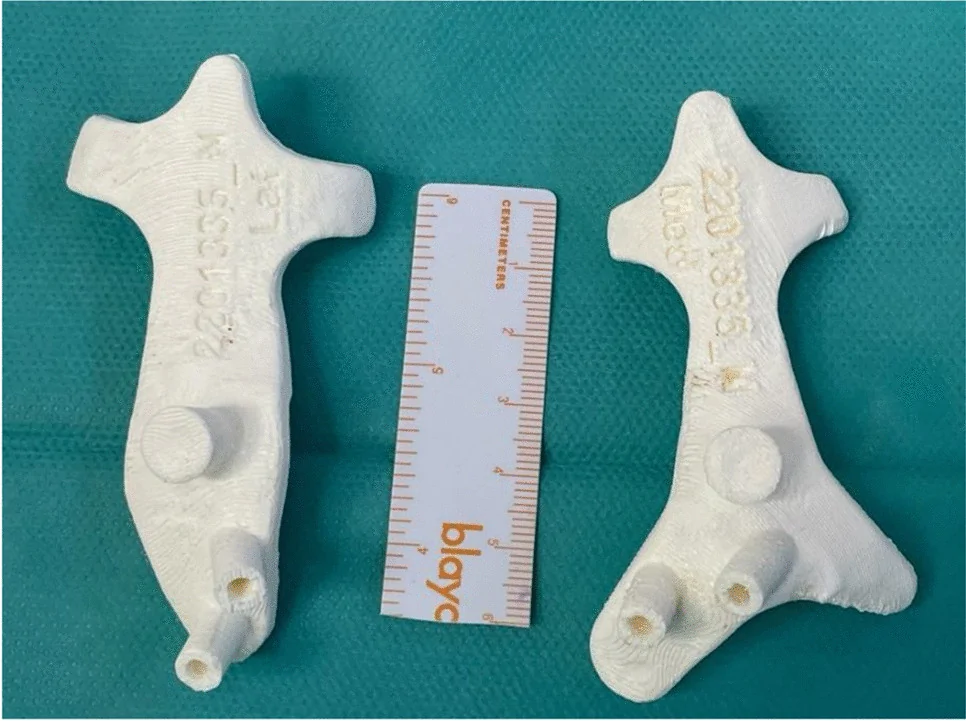
Size of lateral and medial PSI templates

A longitudinal anteromedial incision of approximately 5 cm was made over the medial epicondyle. The crural fascia was exposed, and a longitudinal incision was made down the fascia. Once the medial femoral epicondyle was exposed, the attachments of the adductor magnus tendon, MCL, and POL were identified. Subperiosteal dissection was performed. Once the PSI was adapted to the bone surface, Kirschner wires were inserted. After removing the guide, bone tunnels were made by a 7-mm drill at a 25 mm in depth, as recommended by some authors [27] (Figs. 2 and 3).

### Accuracy analysis

**Fig. 4:**
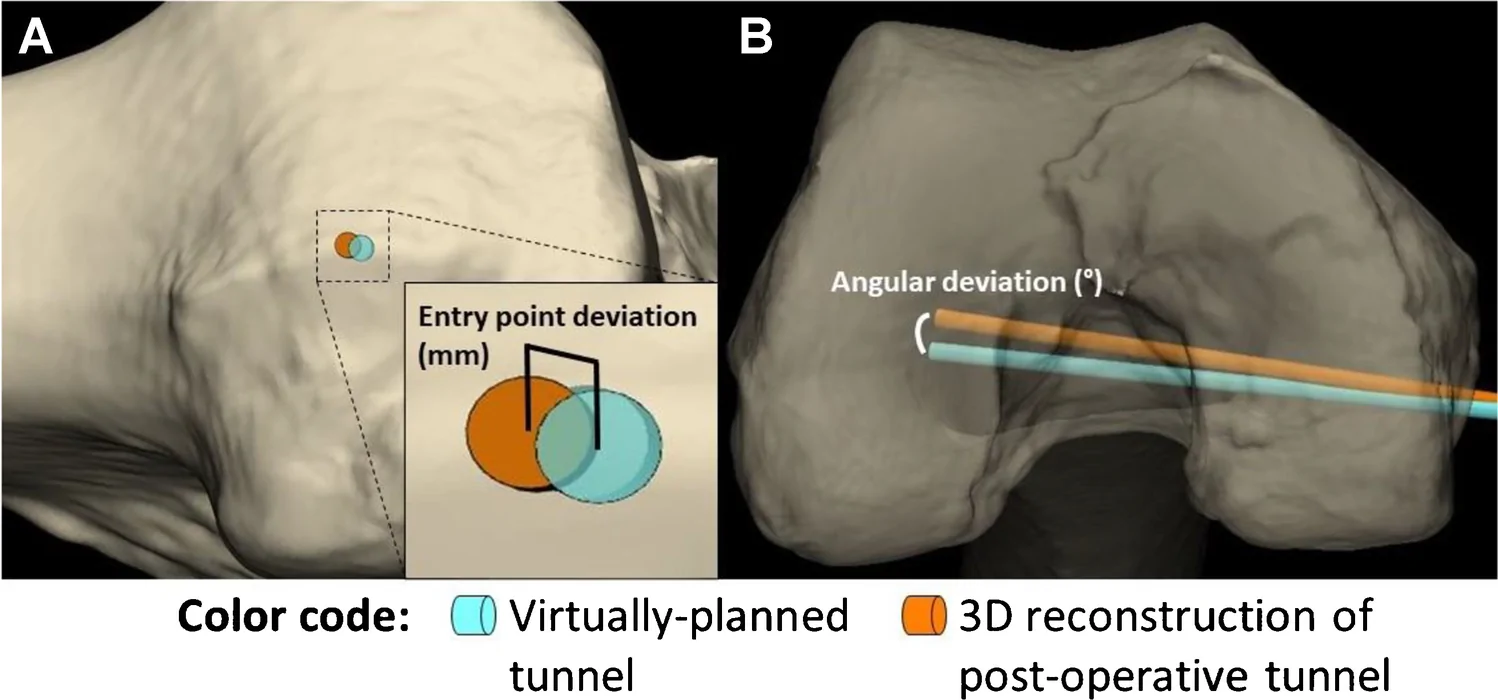
**A**–**B** Superimposition of 3D reconstruction of pre- and post-operative CT scans. Measurements of **A** entry point deviation and **B** angular deviation between planned (blue) and actual (orange) tunnels

**Fig. 5:**
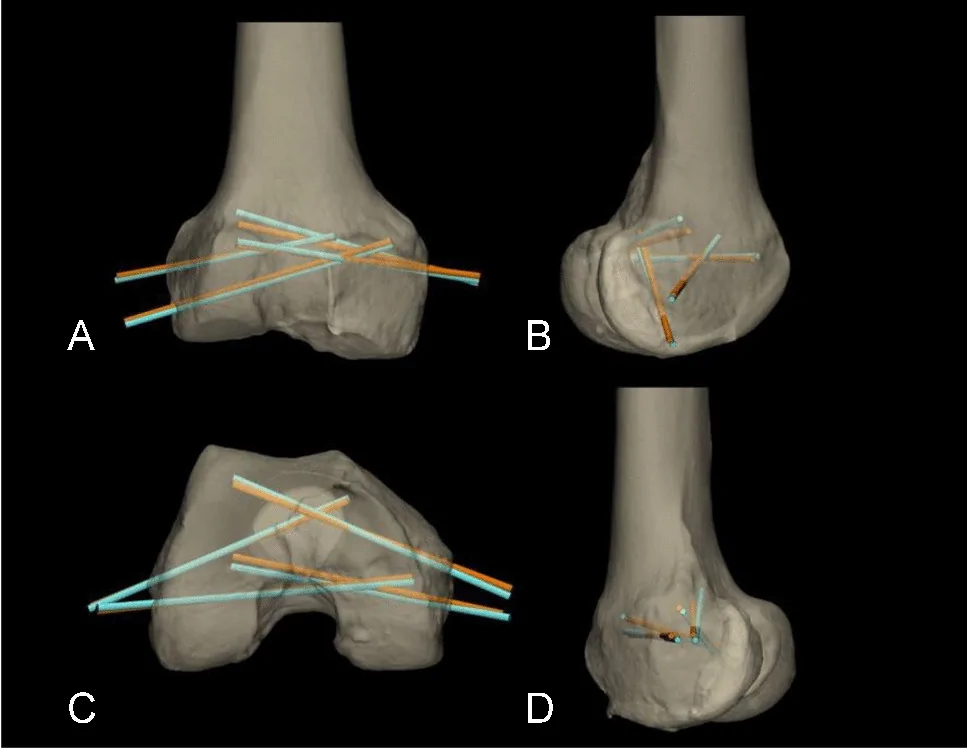
Superimposition of 3D reconstruction of pre- and post-operative CT scans. **A** Coronal, **B** lateral, **C** axial, and **D** medial view. Angular deviation between planned (blue) and actual (orange) tunnels

Post-operative CT scans were performed of each cadaveric knee, followed by segmentation and the creation of mesh-volume files similar to the pre-operative procedure. The accuracy of the use of 3D-printed PSI was assessed by superimposing post-operative CT mesh-volume files onto pre-operative ones. The entry point deviations of the performed tunnels were analyzed from the planned tunnels, measured in millimeters (mm). Then, the angular deviation was analyzed and measured in degrees. Angular deviation is defined as the angle between the vectors crossing from the centres of the planned and performed tunnels in the x, y, and z planes, as shown in Figs. 4A–B and 5A–D.

### Statistical analysis

For all continuous data, the median was used as the central tendency measure, and interquartile ranges (Q1-Q3) were used as the measure of variance. For comparing variables among groups, we used the Kruskal–Wallis test, with *p*-values < 0.05 counting as significant.

## Results

All surgical guides were properly fitted to the corresponding anatomical area. Tables 1 and 2 shows the results of cortical entry point deviation and angular deviation between planned and post-surgical tunnels. Notably, all tunnels were located inside the bone, with no intra-articular invasion. In addition, no tunnel convergences were found. When comparing the variables among the bone tunnels, no statistically significant differences were found, meaning that accuracy levels were similar in all groups analyzed.

**Table 1:** Cortical entry point deviation between planned and post-surgical tunnels

Tunnels	Entry point deviation in (mm)^a^		*p*-Value^b^
	Median	Q1-Q3	
LCL	1.88	0.91–3.11	0.407
PT	2.93	2.33–3.50	
MCL	1.93	1.42–5.68	
POL	2.16	1.21–3.60	

^a^Continuous variables are reported as median values and their corresponding interquartile range (Q1-Q3). ^b^Kruskal-Wallis test. ^*^Differences with a *p*-value of less than 0.05 were considered statistically significant

**Table 2:** Cortical entry point and angular deviation between planned and postsurgical tunnels

Tunnels	Angular deviation in (°)^a^		*p*-Value^b^
	Median	Q1-Q3	
LCL	2.42	1.18–7.67	0.479
PT	4.15	1.79–8.47	
MCL	4.50	2.75–9.09	
POL	4.69	3.81–6.91	

^a^Continuous variables are reported as median values and their corresponding interquartile range (Q1-Q3)

^b^Kruskal-Wallis test

^*^Differences with a *p*-value of less than 0.05 were considered statistically significant

## Discussion

The most important finding of this study was the accuracy observed in the direction of the bone tunnels between those planned with the computer software and the ones that were performed in the cadaveric knees using the custom designed 3D-printed PSI. To avoid intra-operative and post-operative complications, some authors have studied the most suitable angulations required for femoral tunnels in these complex surgeries, but currently, there is no clear consensus on this topic. To perform lateral reconstruction, Moatsche et al. recommended in a descriptive laboratory study an anterior angulation of 35° and 0° in the sagittal and axial planes, respectively, in the LCL and PT [21]. However, Gelber et al. described the safest angulations applying an anterior angulation of 30° on the axial plane and 0° on the coronal plane for the LCL and 30° in the axial and coronal planes for the PT tunnel [19]. When the injury involves the medial corner, Moatsche et al. recommended an anterior angulation of 20–40° and 40° proximally for the MCL in the sagittal and axial planes, respectively, and an anterior angulation of 20° and 20° proximally for the POL in the sagittal and axial planes [21]. Nevertheless, Gelber et al. found that an anterior and proximal angulation of 30° for the MCL and for the POL was the safest direction [20]. Furthermore, all these recommendations may be valid only in patients without previous surgery or in the absence of hardware devices in the distal femur.

Regarding the reconstruction of other knee ligaments, similar studies have determined the optimal angulation for anterior cruciate ligament (ACL) and anterolateral ligament (ALL) tunnel reconstructions in order to avoid coalescence between them in inside-out reconstruction techniques [28, 29]. For extra-articular reconstruction of the ALL in conjunction with ACL reconstruction, two different tunnels must be performed, which have similar risk of coalescence than the bone tunnels in multiligamentary reconstructions. For this purpose, Stodeur et al. recommended an angulation of 40° anterior in the axial plane and 10° proximal in the coronal plane for the anterolateral tunnel [28].

Since no specific tool currently exists to drill bone tunnels with precision, in this scenario, both the entry point and the bone tunnel direction are usually performed freehanded without any previous planning. Then, the success of this technique still depends on the surgeon’s experience. In addition, the aforementioned studies often make angular recommendations taking into account two planes of space, which is more feasible in clinical practice but probably less accurate than a 3D assessment. No studies have been published using this technology to perform complex knee ligament reconstructions. In recent years, PSI has been successfully applied to different areas in orthopaedics and trauma to improve the accuracy of different procedures, such as total knee replacement (TKR) [30, 31], upper extremity fractures [32, 33], or some other procedures showing similar results [34–36]. Differences of approximately 5° in the tunnel angle deviation from planning do not have a relevant surgical repercussion when performing bone tunnels. Therefore, this technology can be useful in managing these complex injuries.

The second important finding of this study was the degree of accuracy found for all bone tunnel entry points, which after surgery was deviated approximately 2 mm as compared to 3D surgical planning. This small deviation would have no clinical repercussions. In this sense, when a conventional technique is used, the surgeon usually has to intra-operatively decide on the entry points based on the anatomical attachments of the ligaments, and many times, this manoeuvre may be difficult due to the absence or sometimes malposition of the injured ligaments [26, 27]. This technique allows us to devise a pre-operative plan so that we can be more accurate during the surgical procedure.

Some limitations have been recognized in this study. In spite of using a minimally invasive approach, avoiding an excessive detachment of soft tissues and using a low profile of PSI, repercussions on soft tissue morbidity were not evaluated when the surgical approach to adapt the designed guide to the femoral bone surface was used. However, this was not the main purpose of the study, and this point may be interesting to evaluate in a clinical trial. Second, no researchers have evaluated the precision of an expert surgeon making all these bone tunnels at a proper entry point and in the proper direction during these surgical procedures. Then, regarding our study design, further studies are needed to conclude that 3D printing technology is more accurate than conventional surgery performed by an expert surgeon. Third, intra-articular bone tunnels were not associated to simulate an anterior or posterior cruciate ligament reconstruction in our study. This may still be a limitation to strongly recommend using this technology to treat multiligamentary injuries in real patients. Last, the sample size, which was based on those of other studies using the same number of cadaveric pieces [19, 20], may have been limited, but it was large enough to analyze accuracy.

Overall, evaluating these results, one of the potential advantages of the use of a specific 3D-printed PSI in the treatment of these lesions is that it may be a good technique to avoid intra-operative and post-operative complications and for managing complex revision cases that include previous hardware or bone tunnels.

## Conclusions

The use of 3D-printed PSI for femoral bone tunnel drilling in multiple-ligament knee injuries provides accurate and reproducible results and may be a promising tool for use in clinical practice.

## Data Availability

Raw data were generated at 3D Surgical Planning Lab at Hospital Parc Taulí. Derived data supporting the findings of this study are available from the corresponding author FFG on request.
